# Effects of Tirzepatide on Pancreatic β-Cell Function in Adults With Type 2 Diabetes Mellitus: A Systematic Review

**DOI:** 10.7759/cureus.116733

**Published:** 2026-09-22

**Authors:** Rasha Mahmoud, Ahmed Abdalla Zarrouq Yousif, Alaa Fathelrahman Mokhtar Osman, Eltayeb Osman Elfaki Omer, Ayaa Ismail Ahmed Talaat, Omima Mohamed, Shereen Alnagi Mohammed Akasha, Emad Salah Fathy Saleh, Hanady ME M Osman

**Affiliations:** 1 Ophthalmology, University of Khartoum, Khartoum, SDN; 2 Endocrinology, Sligo University Hospital, Sligo, IRL; 3 Internal Medicine, University Hospital Kerry, Tralee, IRL; 4 Endocrinology, Dr. Sulaiman Al Habib Hospital, Riyadh, SAU; 5 General Medicine, Ahfad University for Women, Khartoum, SDN; 6 General Medicine, Bon Secours Hospital Limerick, Limerick, IRL; 7 Internal Medicine, Bupa Care Connect, Jeddah, SAU; 8 General Medicine, Nizwa Hospital, Nizwa, OMN; 9 Quality Improvement and Patient Safety, Najran Armed Forces Hospital, Ministry of Defense Health Services, Najran, SAU

**Keywords:** disposition index, dual gip/glp-1 receptor agonist, homa2-b, pancreatic β-cell function, systematic review, tirzepatide, type 2 diabetes mellitus

## Abstract

Type 2 diabetes mellitus (T2DM) is driven by progressive pancreatic β-cell failure, and few glucose-lowering agents demonstrably modify this defect. This systematic review assessed whether tirzepatide, a dual glucose-dependent insulinotropic polypeptide (GIP) and glucagon-like peptide-1 (GLP-1) receptor agonist, improves β-cell function in adults with T2DM. Following the Preferred Reporting Items for Systematic Reviews and Meta-Analyses (PRISMA) 2020 statement, PubMed, Web of Science, Scopus and Embase were searched from inception to July 29, 2026, with citation searching. Eligible reports were original peer-reviewed primary human studies reporting a quantitative measure of β-cell function in adults receiving subcutaneous tirzepatide; preclinical and non-original reports were excluded. Risk of bias was appraised with risk of bias 2 (RoB 2) tool and The Risk Of Bias In Non-randomized Studies of Interventions, Version 2 (ROBINS-I). Heterogeneity of outcome metrics precluded meta-analysis, so findings were synthesised narratively. Eight reports representing six distinct populations and 3,442 unique participants were included. Tirzepatide 5-15 mg weekly raised the Homeostasis Model Assessment 2 of Beta-cell Function (HOMA2-B) by 77% to 163% from baseline, exceeding placebo, dulaglutide and semaglutide 1 mg; fasting intact proinsulin and the intact proinsulin/C-peptide ratio fell by up to 55% and 49%; and the clamp disposition index rose approximately six-fold. Fasting insulin and C-peptide fell in parallel, indicating reduced secretory demand. Risk of bias was low in four reports, of some concern in one, high in two and serious in one. Tirzepatide improves β-cell function indices beyond selective GLP-1 receptor agonism, but the evidence rests largely on post hoc, industry-funded analyses and no study examined function after drug withdrawal. This review was unfunded and unregistered.

## Introduction and background

Diabetes mellitus is now among the most prevalent chronic diseases worldwide. The 11th edition of the International Diabetes Federation Diabetes Atlas estimated that 589 million adults aged 20 to 79 years, or 11.1% of that age group, were living with diabetes in 2024, that 252 million of them were unaware of the diagnosis, and that the total will rise to approximately 853 million by 2050, an increase of 45% against projected population growth of 25%; type 2 diabetes mellitus (T2DM) accounts for the large majority of this burden [1]. T2DM is not a static disorder of glucose transport but a progressive disease in which the capacity of the pancreatic β-cell to secrete insulin appropriately for the prevailing metabolic load declines relentlessly over time. The United Kingdom Prospective Diabetes Study established this trajectory unequivocally, demonstrating that β-cell function continued to deteriorate over six years irrespective of whether patients were randomised to diet, sulphonylurea or insulin, so that secondary failure of monotherapy became the expected rather than the exceptional outcome [2]. Subsequent pathophysiological synthesis broadened the framework beyond the classical duo of insulin resistance and impaired secretion to encompass at least eight interacting defects, including excessive glucagon release from the pancreatic α-cell, a blunted incretin effect, accelerated lipolysis and increased renal glucose reabsorption [3]. Because most conventional glucose-lowering agents reduce glycated haemoglobin (HbA1c) without demonstrably altering the underlying secretory lesion, therapies that measurably improve β-cell function are of substantial clinical and mechanistic interest.

Tirzepatide is a 39-amino-acid synthetic peptide engineered from the glucose-dependent insulinotropic polypeptide (GIP) sequence and modified with a C20 fatty di-acid moiety that permits once-weekly subcutaneous administration; it acts as an agonist at both the GIP and the glucagon-like peptide-1 (GLP-1) receptors, with greater relative affinity for the GIP receptor [4]. This dual mechanism was pursued on the hypothesis that GIP receptor agonism would add metabolic benefit to the established actions of selective GLP-1 receptor agonists. The phase 3 SURPASS programme demonstrated that tirzepatide monotherapy in participants with early T2DM reduced glycated haemoglobin (HbA1c) from baseline by up to 2.07%, against a 0.04% increase on placebo, with body weight reductions of up to 9.5 kg [5], and that in participants inadequately controlled on metformin it was superior to semaglutide 1 mg for both HbA1c and weight across all three maintenance doses [6]. The magnitude of glycaemic normalisation achieved, with a substantial proportion of participants attaining an HbA1c below the diagnostic threshold for diabetes, raises the central mechanistic question of whether these effects arise principally from weight loss and improved insulin sensitivity, or whether they additionally reflect a direct and quantifiable restoration of β-cell secretory competence.

Answering that question requires attention to how β-cell function is measured. Fasting surrogate indices derived from the updated homeostatic model, most commonly the Homeostasis Model Assessment 2 of Beta-cell Function (HOMA2-B) computed from paired fasting glucose and either insulin or C-peptide using the validated computer program rather than the superseded linear approximation, are inexpensive and scalable but capture only basal secretion referenced to prevailing glycaemia [7]. Markers of insulin processing such as fasting intact proinsulin and the proinsulin-to-C-peptide ratio provide complementary insight into β-cell secretory stress, while dynamic assessments using mixed-meal tolerance testing and gold-standard clamps, yielding the insulin secretion rate, β-cell glucose sensitivity and the disposition index, characterise stimulated secretory capacity adjusted for ambient insulin sensitivity. Individual analyses of tirzepatide have reported each of these classes of outcomes, but they are dispersed across mechanistic, phase 2 and phase 3 literature, employ different comparators and follow-up durations, and have not previously been assembled and critically appraised in a single evidence synthesis.

The primary objective of this systematic review was to determine the direction and magnitude of change in quantitative measures of pancreatic β-cell function in adults with T2DM treated with subcutaneous tirzepatide. Four secondary objectives were specified: to compare those changes against placebo and against active glucose-lowering comparators; to examine dose-response across the 5, 10 and 15 mg maintenance doses; to describe concurrent changes in α-cell function, insulin sensitivity, glycaemia and body weight in the same participants; and to appraise the risk of bias and the methodological character of the available evidence. Preclinical, animal and in vitro investigations were not eligible for the synthesis, since the review question concerned quantified β-cell outcomes in humans, but the mechanistic literature is considered in the Discussion where it bears on interpretation.

## Review

Materials and methods

Protocol and Reporting

This review was designed, conducted and reported in accordance with the Preferred Reporting Items for Systematic Reviews and Meta-Analyses (PRISMA) 2020 statement and its 27-item checklist [8]. A written protocol specifying the research question, eligibility criteria, search strategy, data items and risk-of-bias approach was prepared before the searches were executed, and no amendments were made to the eligibility criteria thereafter. The protocol was not prospectively registered, which is acknowledged in the Limitations. Because only aggregate published data were used, ethical approval and informed consent were not required.

Eligibility Criteria

Eligibility was defined a priori using the Population, Intervention, Comparator, Outcomes and Study design (PICOS) framework, which structures the review question and improves the specificity of literature searching where resources are limited [9]. The full criteria are presented in Table 1. In summary, eligible studies enrolled adults aged 18 years or older with T2DM, administered subcutaneous tirzepatide at any dose, used any comparator or none, and reported at least one quantitative measure of pancreatic β-cell function. Only original peer-reviewed primary research in human participants was eligible. No restriction was applied by publication year or region; because fewer than 10 eligible studies were identified, the prespecified contingency to restrict inclusion to the preceding five years was not invoked.

**Table 1:** Eligibility criteria structured by the PICOS framework BMI: Body mass index; HbA1c: glycated haemoglobin; HOMA-B/HOMA2-B: homeostatic model assessment index of β-cell function; T2DM: type 2 diabetes mellitus; PICOS: Population, Intervention, Comparator, Outcomes and Study design

Domain	Included	Excluded
Population	Adults ≥18 years with type 2 diabetes mellitus; any diabetes duration, HbA1c, BMI or background therapy	Type 1, monogenic or secondary diabetes; prediabetes or obesity without diabetes; pregnancy; age <18 years
Intervention	Subcutaneous tirzepatide, any dose (2.5-15 mg) and duration, as monotherapy or add-on	Oral tirzepatide; other incretin co-agonists studied without a tirzepatide arm
Comparator	Placebo, any active glucose-lowering comparator, usual care, or none (single-arm designs)	None; absence of a comparator was not grounds for exclusion
Outcomes	At least one quantitative measure of β-cell function: HOMA-B or HOMA2-B; proinsulin or proinsulin ratios; fasting or stimulated C-peptide and insulin; insulin secretion rate; β-cell glucose sensitivity; insulinogenic or disposition index	Reports of glycaemic, anthropometric, lipid or safety outcomes only, with no measure of β-cell function
Study design	Original peer-reviewed primary research in humans: randomised trials, their prespecified secondary or post hoc analyses, non-randomised interventional studies, observational cohorts	Reviews, meta-analyses, editorials, letters, conference abstracts, protocols, preprints, case reports; preclinical, animal and in vitro studies
Reports	Full text available; sufficient numerical detail to characterise the β-cell outcome; no date or country restriction	Non-English full texts; full text unobtainable; duplicate reports of an already-included analysis

Information Sources and Search Strategy

PubMed, Web of Science Core Collection, Scopus and Embase were searched from database inception to July 29, 2026. ClinicalTrials.gov, the European Union Clinical Trials Register and the University Hospital Medical Information Network Clinical Trials Registry were interrogated to identify completed trials whose results might not have been captured in the indexed literature. The reference lists of all included studies and of relevant reviews were hand-searched, and forward citation tracking of the key mechanistic reports was performed in Google Scholar. The complete database-specific strategies are given in Table 2.

**Table 2:** Database-specific search strategies MeSH: Medical Subject Headings; GIP: glucose-dependent insulinotropic polypeptide; GLP-1: glucagon-like peptide-1; HOMA: homeostatic model assessment. The searches were executed on July 29, 2026 with no publication-date or language limit.

Database	Platform and fields	Complete search strategy
PubMed	PubMed; MeSH, Supplementary Concept and title/abstract fields	("tirzepatide"[Supplementary Concept] OR tirzepatide[tiab] OR LY3298176[tiab] OR Mounjaro[tiab] OR Zepbound[tiab] OR "dual GIP and GLP-1 receptor agonist"[tiab] OR twincretin[tiab]) AND ("Insulin-Secreting Cells"[Mesh] OR "beta cell"[tiab] OR "beta cells"[tiab] OR "β-cell"[tiab] OR "β-cells"[tiab] OR "beta-cell function"[tiab] OR "islet function"[tiab] OR HOMA*[tiab] OR "HOMA2-B"[tiab] OR "HOMA-B"[tiab] OR proinsulin[tiab] OR "C-peptide"[tiab] OR "insulin secretion"[tiab] OR "insulin secretion rate"[tiab] OR "disposition index"[tiab] OR "insulinogenic index"[tiab] OR "glucose sensitivity"[tiab] OR "hyperglycemic clamp"[tiab] OR "hyperglycaemic clamp"[tiab]) AND ("Diabetes Mellitus, Type 2"[Mesh] OR "type 2 diabetes"[tiab] OR T2D[tiab] OR T2DM[tiab] OR "type II diabetes"[tiab])
Embase	Embase; Emtree terms and free-text fields	('tirzepatide'/exp OR tirzepatide:ti,ab,kw OR LY3298176:ti,ab,kw OR Mounjaro:ti,ab,kw OR Zepbound:ti,ab,kw OR 'dual GIP and GLP-1 receptor agonist':ti,ab,kw OR twincretin:ti,ab,kw) AND ('beta cell'/exp OR 'beta cell function':ti,ab,kw OR 'beta-cell function':ti,ab,kw OR 'islet function':ti,ab,kw OR HOMA*:ti,ab,kw OR 'HOMA2-B':ti,ab,kw OR 'HOMA-B':ti,ab,kw OR proinsulin:ti,ab,kw OR 'C-peptide':ti,ab,kw OR 'insulin secretion':ti,ab,kw OR 'insulin secretion rate':ti,ab,kw OR 'disposition index':ti,ab,kw OR 'insulinogenic index':ti,ab,kw OR 'glucose sensitivity':ti,ab,kw OR 'hyperglycemic clamp':ti,ab,kw OR 'hyperglycaemic clamp':ti,ab,kw) AND ('type 2 diabetes mellitus'/exp OR 'type 2 diabetes':ti,ab,kw OR T2D:ti,ab,kw OR T2DM:ti,ab,kw OR 'type ii diabetes':ti,ab,kw)
Scopus	Scopus; TITLE-ABS-KEY	TITLE-ABS-KEY((tirzepatide OR LY3298176 OR Mounjaro OR Zepbound OR "dual GIP and GLP-1 receptor agonist" OR twincretin) AND ("beta cell" OR "beta cells" OR "β-cell" OR "β-cells" OR "beta-cell function" OR "islet function" OR HOMA* OR "HOMA2-B" OR "HOMA-B" OR proinsulin OR "C-peptide" OR "insulin secretion" OR "insulin secretion rate" OR "disposition index" OR "insulinogenic index" OR "glucose sensitivity" OR "hyperglycemic clamp" OR "hyperglycaemic clamp") AND ("type 2 diabetes" OR T2D OR T2DM OR "type II diabetes"))
Web of Science Core Collection	Web of Science; Topic (TS) field	TS=((tirzepatide OR LY3298176 OR Mounjaro OR Zepbound OR "dual GIP and GLP-1 receptor agonist" OR twincretin) AND ("beta cell" OR "beta cells" OR "β-cell" OR "β-cells" OR "beta-cell function" OR "islet function" OR HOMA* OR "HOMA2-B" OR "HOMA-B" OR proinsulin OR "C-peptide" OR "insulin secretion" OR "insulin secretion rate" OR "disposition index" OR "insulinogenic index" OR "glucose sensitivity" OR "hyperglycemic clamp" OR "hyperglycaemic clamp") AND ("type 2 diabetes" OR T2D OR T2DM OR "type II diabetes"))

No language filter was applied within any database interface, so non-English records were retrieved and screened alongside the remainder. The restriction to English-language reports operated only at the full-text eligibility stage. No record was excluded on language grounds: every full text assessed for eligibility was available in English, and the 17 exclusions at that stage were made on population and publication-type criteria. The language restriction therefore did not alter the set of studies included in this review, although it remains a potential source of bias for future updates and is retained in the Limitations.

Study Selection and Safeguards Against Selection Bias

Records were exported to a reference manager, deduplicated electronically and verified manually. Titles and abstracts were screened against the written eligibility criteria, the full texts of all potentially eligible records were assessed, and each excluded full text was assigned a single primary reason for exclusion; the list of excluded full texts with reasons is available from the corresponding author.

Screening for this review was not performed in duplicate independently, and several safeguards were therefore applied to limit the resulting risk of selection bias. Eligibility criteria were fixed in writing before the search was run and were applied unchanged throughout. Title-and-abstract screening and full-text assessment were each performed twice, the second pass at least two weeks after the first and without reference to the earlier decisions, and any record classified differently between the two passes was resolved by re-reading the full text against the written criteria. The search was re-run before analysis to capture newly indexed records, and reference-list hand-searching and forward citation tracking were used as independent routes to identification, so that a study missed at title-and-abstract screening had two further opportunities to be retrieved; three of the eight included studies were in fact identified through citation searching rather than through the database searches. Extracted data were checked against the source publication in a separate verification pass, and every numerical value appearing in the tables was traced back to the specific published table or sentence from which it was taken. These procedures reduce but do not replace independent duplicate screening, and the residual limitation is stated explicitly in the Limitations.

Data Items and Outcome Definitions

Data were extracted into a piloted standardised spreadsheet capturing first author, publication year, country and setting, study design and parent trial, registration identifier, population and eligibility criteria, sample size, tirzepatide dose and titration schedule, comparator, background glucose-lowering therapy, treatment duration and analysis population.

The primary data items were quantitative measures of β-cell function, each recorded together with the method by which it was derived, because these measures are not interchangeable. Fasting model-derived indices were recorded as HOMA2-B or HOMA2-%B with the substrate used for computation, insulin or C-peptide, specified, since the two are not numerically equivalent and are reported separately throughout this review. Insulin-processing markers comprised fasting intact proinsulin and the proinsulin-to-C-peptide and proinsulin-to-insulin ratios. Dynamic measures comprised parameters derived from mixed-meal tolerance testing, including areas under the concentration-time curve and model-derived insulin secretion rates and β-cell glucose sensitivity, together with parameters derived from hyperglycaemic and hyperinsulinaemic-euglycaemic clamps, including first-phase, second-phase and total insulin secretion rate, the M-value, the glucose infusion rate and the disposition index. Assay and clamp protocols were recorded as reported by the source studies; measurement was laboratory-based in every included report and no study was excluded on assay grounds. Ancillary items were α-cell measures, insulin-sensitivity indices, adipokines, glycaemic and anthropometric outcomes, and the authors’ stated conclusions. Where several reports described one parent trial, they were linked so that participants were not double-counted, and the unique participant total therefore reflects distinct study populations rather than the sum of individual sample sizes. Percentage changes are reported as published; no value was back-calculated or imputed.

Risk-of-Bias Assessment

Reports of randomised trials, including prespecified secondary and post hoc analyses, were appraised at the outcome level with the revised Cochrane risk-of-bias tool for randomised trials (RoB 2) across its five domains [10]. Because β-cell function was rarely a prespecified primary endpoint, particular weight was given to the domain concerning selection of the reported result. The non-randomised single-arm interventional study was appraised with the Risk Of Bias In Non-randomised Studies of Interventions (ROBINS-I) tool across its seven domains, with the absence of a concurrent control and the co-occurrence of weight loss and background metformin treated as the principal confounding concerns [11]. Domain-level and overall judgements for both tools were summarised graphically using robvis (Risk-Of-Bias VISualization), a Shiny web application that generates publication-quality traffic-light plots from RoB 2 and ROBINS-I assessments [12].

Data Synthesis and Statistical Approach

Quantitative meta-analysis was not undertaken. The included reports used substantially different metrics of β-cell function, different comparators and follow-up periods ranging from 12 to 52 weeks, and several described overlapping participants from the same parent trial; pooling across these dimensions would have produced a statistically precise but clinically uninterpretable summary estimate. The results were therefore synthesised narratively, grouped by class of β-cell measure, with effect direction, magnitude, dose-response and consistency across populations examined.

Because no pooled estimate was generated, no measure of statistical heterogeneity such as I² or τ² was computed, no meta-regression or sensitivity meta-analysis was performed, and no funnel plot or statistical test for small-study effects was applied; each of these procedures presupposes a pooled analysis and would be uninterpretable across eight reports using non-equivalent outcome metrics. Effect estimates are reported as published, with the comparator value, the estimated treatment difference and its 95% confidence interval wherever the source study provided them. P values are reproduced as stated by the original authors and are interpreted throughout as nominal, because none of the included analyses adjusted for multiplicity. The certainty of the body of evidence was not formally graded with the Grading of Recommendations, Assessment, Development and Evaluation (GRADE) framework, since several GRADE domains are defined with reference to a pooled effect estimate; the risk-of-bias appraisal and the explicit appraisal of consistency, dose-response and directness in the Discussion serve this function instead. No additional statistical review is considered necessary, as the review contains no inferential statistics generated by the authors.

Results

Study Selection

Database searching identified 253 records (PubMed n=96; Web of Science n=56; Scopus n=62; Embase n=39) and citation searching identified three further relevant studies. After removal of 134 duplicates, 119 records were screened by title and abstract and 94 were excluded. Of the 25 reports sought for retrieval, three could not be obtained, leaving 22 assessed in full text. Seventeen were excluded, 14 because the study population was not adults with T2DM and three because they were review articles, editorials or abstracts. Five studies were included from database searching and three from citation searching, giving eight included reports [13-20] (Figure 1). These represented six distinct study populations comprising 3,442 unique participants: two reports [14,18] presented complementary analyses of the same phase 1 mechanistic trial, and one report [15] described a pharmacodynamic substudy nested within the trial reported by another [19].

**Figure 1:**
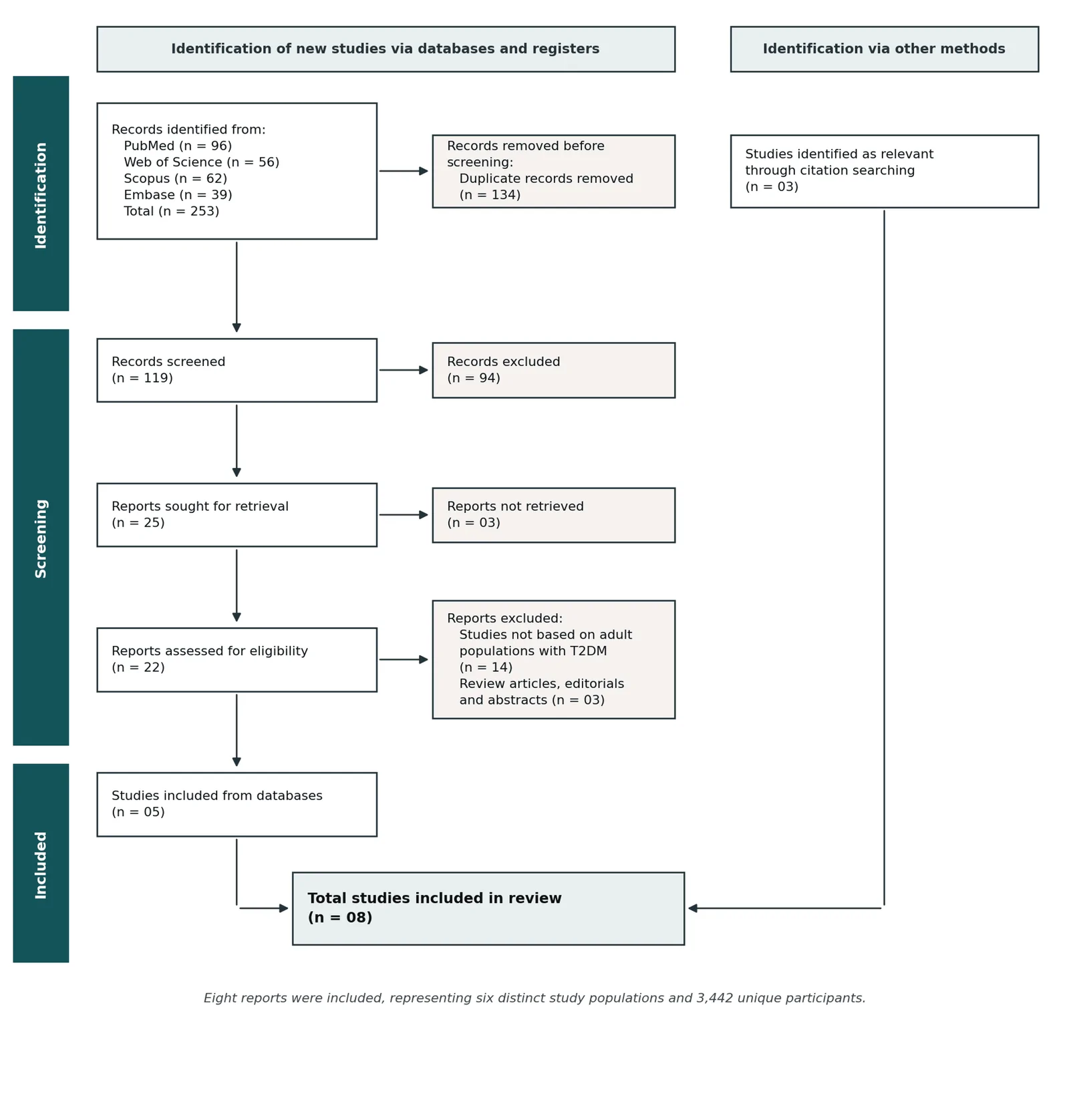
PRISMA 2020 flow diagram of study identification, screening and inclusion PRISMA: Preferred Reporting Items for Systematic Reviews and Meta-Analyses; T2DM: type 2 diabetes mellitus.

Characteristics of the Included Studies

Seven included reports were analyses of randomised controlled trials [13-19] and one was a prospective single-arm interventional study [20]; their characteristics are given in Table 3. Three were conducted in Japan [15,19,20], two in Germany [14,18] and three in multinational settings [13,16,17]. Sample sizes ranged from 16 to 1,879 participants and treatment duration from 12 to 52 weeks. Tirzepatide was given once weekly at maintenance doses of 5, 10 and 15 mg in five reports [13,15-17,19], at 15 mg alone in the two reports of the phase 1 trial [14,18], and to a maximum of 5 mg in the short Japanese clamp study [20]. Background therapy differed materially: two analyses studied monotherapy after washout or in drug-naive participants [16,19], three studied tirzepatide added to metformin [14,17,18], and the remainder permitted mixed backgrounds. Populations were middle-aged, with mean diabetes duration between 5.9 and 8.6 years and mean baseline HbA1c between approximately 7.95% and 8.3%. Mean body mass index differed appreciably by region, from 27.5 to 28.1 kg/m² in the Japanese trials to 34.2 kg/m² in SURPASS-2, which is directly relevant to the interpretation of insulin sensitivity outcomes.

**Table 3:** Characteristics of the eight included studies BMI: body mass index; HbA1c: glycated haemoglobin; QW: once weekly; RCT: randomised controlled trial; T2DM: type 2 diabetes mellitus. Reports [14] and [18] analyse the same phase 1 trial, and [15] is a substudy nested within the trial reported in [19].

Study [ref]	Country	Design (parent trial)	Population	N	Tirzepatide	Comparator	Duration
Thomas et al. 2021 [13]	Multinational	Post hoc biomarker analysis of a phase 2b double-blind RCT	T2DM on diet and exercise or metformin	316	1, 5, 10, 15 mg QW	Dulaglutide 1.5 mg; placebo	26 weeks
Heise et al. 2022 [14]	Germany	Phase 1 double-blind mechanistic clamp RCT (NCT03951753); β-cell endpoint primary	T2DM ≥ 6 months on metformin ± one oral agent	117	15 mg QW	Semaglutide 1 mg; placebo	28 weeks
Yabe et al. 2023 [15]	Japan	Prespecified pharmacodynamic substudy of SURPASS J-mono (NCT03861052)	Japanese adults, oral-agent naive or after washout	48	5, 10, 15 mg QW	Dulaglutide 0.75 mg	52 weeks
Lee et al. 2023 [16]	Multinational	Post hoc biomarker analysis of the phase 3 placebo-controlled SURPASS-1 trial	Early T2DM on diet and exercise alone	478	5, 10, 15 mg QW	Placebo	40 weeks
Frias et al. 2024 [17]	Multinational	Post hoc biomarker and quartile analysis of the phase 3 open-label SURPASS-2 trial (NCT03987919)	T2DM inadequately controlled on metformin ≥1500 mg/day	1,879	5, 10, 15 mg QW	Semaglutide 1 mg	40 weeks
Mather et al. 2024 [18]	Germany	Model-based secondary analysis of meal-test data from the phase 1 trial (NCT03951753)	T2DM on metformin (same cohort as Heise 2022)	117	15 mg QW	Semaglutide 1 mg; placebo	28 weeks
Hamamoto et al. 2025 [19]	Japan	Prespecified secondary and post hoc subgroup analysis of SURPASS J-mono (NCT03861052)	Japanese adults, BMI ≥23, HbA1c 7.0–10.0%	636	5, 10, 15 mg QW	Dulaglutide 0.75 mg	52 weeks
Yamaguchi et al. 2025 [20]	Japan	Prospective single-arm open-label clamp study (UMIN000056862)	Japanese adults with T2DM and obesity (BMI ≥ 25)	16	2.5 → 5 mg QW	None	12 weeks

Fasting Model-derived Indices of β-Cell Function

Five reports quantified β-cell function using fasting model-derived indices (Table 4, Figure 2). HOMA2-B computed with C-peptide rose by 93% to 163% across tirzepatide doses over 26 weeks in the phase 2b analysis, against 72% for dulaglutide 1.5 mg, with all doses from 5 mg upward significantly exceeding placebo (P≤0.02) [13]. In SURPASS-1, where tirzepatide was given as monotherapy in early T2DM, the index rose by 77% to 92% at 40 weeks against a 1.4% decline on placebo (P<0.001) [16]. In SURPASS-2, the increases were 96.9%, 111.5% and 120.4% with 5, 10 and 15 mg, each significantly greater than the 84.0% seen with semaglutide 1 mg (estimated treatment differences 7.0, 15.0 and 19.8) [17]. In SURPASS J-mono at 52 weeks, HOMA2-%B rose by 90.0% to 111.8% (C-peptide-based) and 105.4% to 126.8% (insulin-based) against 44.4% and 61.3% with dulaglutide 0.75 mg, with treatment differences of up to 46.7% (P<0.001) [19]. In the 12-week single-arm study using only the 5 mg dose, HOMA2-%β increased significantly from baseline (P<0.001) [20]. The direction of effect was identical in every study, the effect was dose-dependent wherever multiple doses were tested, and tirzepatide exceeded every active comparator.

**Table 4:** Fasting model-derived indices of β-cell function and markers of insulin processing HOMA: Homeostasis Model Assessment; ETD: estimated treatment difference versus comparator, with 95% confidence intervals in parentheses where reported. Values are least-squares mean percentage changes from baseline as published. Tirzepatide values are given in ascending dose order (5, 10, 15 mg) unless otherwise stated.

Study [ref]	Measure	Tirzepatide	Comparator	Effect estimate	Week
Thomas et al. 2021 [13]	HOMA2-B (C-peptide)	+93% to +163%	Dulaglutide 1.5 mg: +72%	Significant vs placebo at 5, 10, 15 mg (P≤0.02)	26
Thomas et al. 2021 [13]	Proinsulin/C-peptide ratio	Dose-dependent fall, up to ≈50%	Dulaglutide 1.5 mg: smaller fall	Tirzepatide 10 and 15 mg superior to dulaglutide	26
Lee et al. 2023 [16]	HOMA2-B (C-peptide)	+77% to +92%	Placebo: -1.4%	P<0.001, all doses	40
Lee et al. 2023 [16]	Fasting intact proinsulin	-49% to -55%	Placebo: -0.6%	P<0.001, all doses	40
Lee et al. 2023 [16]	Intact proinsulin/C-peptide ratio	-47% to -49%	Placebo: -0.1%	P<0.001, all doses	40
Lee et al. 2023 [16]	Fasting insulin	-2% to -12%	Placebo: +15%	P≤0.031 except 10 mg	40
Frias et al. 2024 [17]	HOMA2-B (C-peptide)	+96.9%, +111.5%, +120.4%	Semaglutide 1 mg: +84.0%	ETD +7.0 (0.7-13.7), +15.0 (8.2-22.2), +19.8 (12.7-27.4)	40
Frias et al. 2024 [17]	Fasting C-peptide	-3.5%, -5.2%, -6.0%	Semaglutide 1 mg: +3.3%	Significant at 10 and 15 mg (P<0.05)	40
Frias et al. 2024 [17]	Fasting insulin	-8.9%, -14.6%, -20.9%	Semaglutide 1 mg: +0.6%	ETD -9.5, -15.1, -21.4 (P<0.05)	40
Hamamoto et al. 2025 [19]	HOMA2-%B (C-peptide)	+90.0% to +111.8%	Dulaglutide 0.75 mg: +44.4%	ETD +31.6%, +40.1%, +46.7% (P<0.001)	52
Hamamoto et al. 2025 [19]	HOMA2-%B (insulin)	+105.4% to +126.8%	Dulaglutide 0.75 mg: +61.3%	ETD +27.3%, +34.9%, +40.6% (P<0.001)	52
Hamamoto et al. 2025 [19]	Fasting insulin/C-peptide	-11.2% to -20.9% / -13.8% to -21.1%	Dulaglutide 0.75 mg: +14.6%/+0.53%	P<0.001, all doses	52
Yamaguchi et al. 2025 [20]	HOMA2-%β	Significant increase	None (single-arm)	P<0.001 vs baseline	12

**Figure 2:**
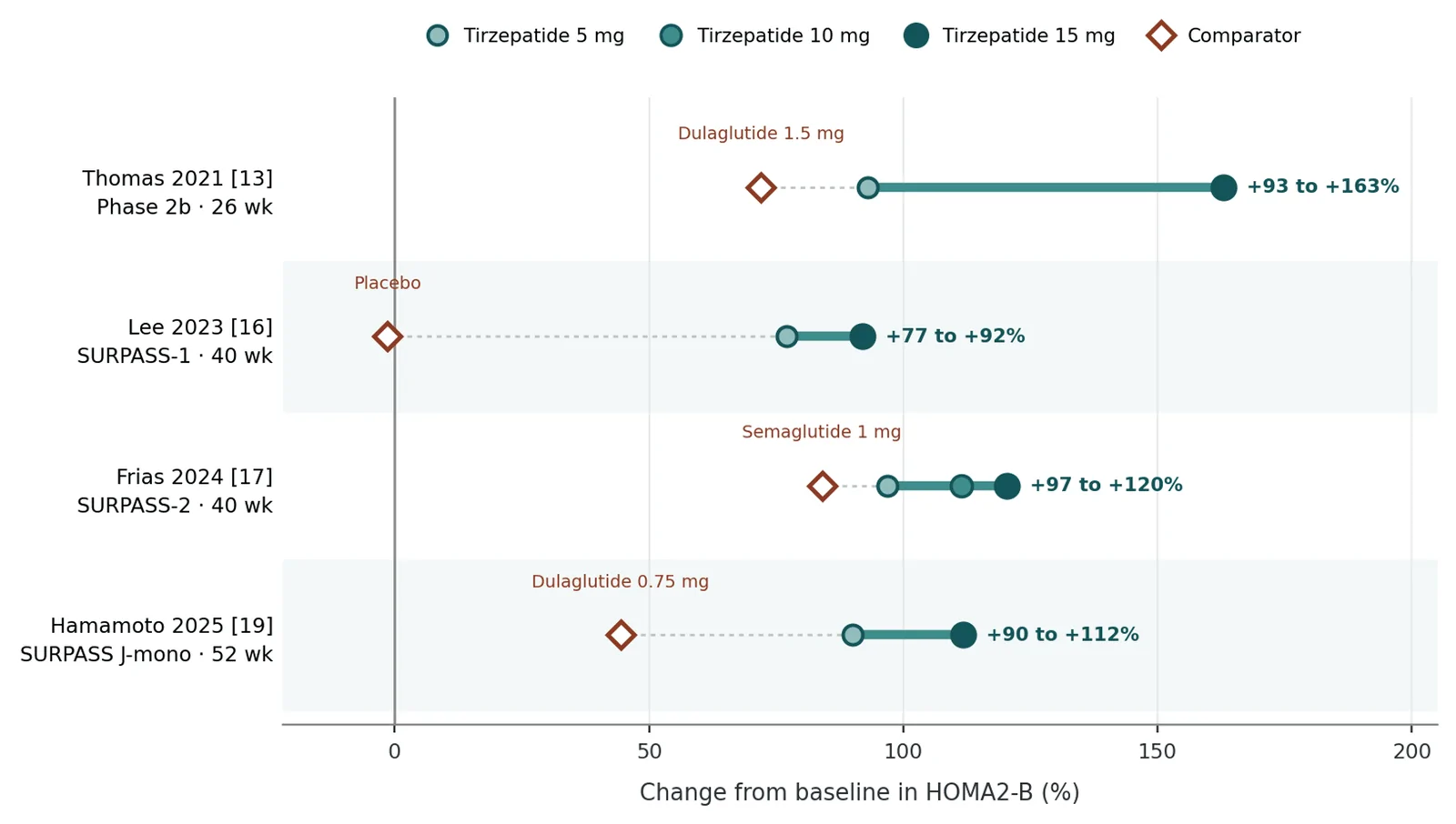
Change from baseline in HOMA2-B with tirzepatide versus comparator All values are HOMA2-B computed with fasting C-peptide. The teal segment spans the lowest to the highest tirzepatide maintenance dose group; only Frias 2024 [17] reported values for each dose separately, so the intermediate 10 mg marker is shown for that study alone. Open diamonds mark the comparator. Data from references [13,16,17,19].

These increases occurred alongside reductions, not increases, in circulating fasting insulin and C-peptide, which is important for their interpretation. Fasting insulin fell by 2% to 12% against a 15% rise on placebo in SURPASS-1 [16], by 8.9% to 20.9% against a 0.6% rise with semaglutide in SURPASS-2 [17], and by 11.2% to 20.9% against a 14.6% rise with dulaglutide in SURPASS J-mono [19]. Fasting C-peptide followed the same pattern, falling by 3.5% to 6.0% in SURPASS-2 [17] and by 13.8% to 21.1% in SURPASS J-mono [19]. In the single-arm clamp study, both were statistically unchanged despite a significant fall in fasting plasma glucose [20].

Markers of Insulin Processing and β-Cell Secretory Stress

Two reports assessed proinsulin-based markers, which index the efficiency of insulin biosynthesis and processing and are regarded as biomarkers of β-cell secretory stress (Table 4). Tirzepatide dose-dependently reduced fasting intact proinsulin and the proinsulin/C-peptide and proinsulin/insulin ratios in the phase 2b analysis, with reductions in the proinsulin/C-peptide ratio of up to approximately 50% and significantly greater reductions than dulaglutide 1.5 mg at the 10 and 15 mg doses [13]. In SURPASS-1, fasting proinsulin fell by 49% to 55% and the intact proinsulin/C-peptide ratio by 47% to 49%, against reductions of 0.6% and 0.1% on placebo (P<0.001) [16]. These findings indicate that improved fasting β-cell function is accompanied by a measurable reduction in the burden placed on the insulin biosynthetic pathway.

Dynamic and Clamp-derived Measures

Four reports provided dynamic or clamp-based assessments (Table 5). The phase 1 mechanistic trial was the only included study in which a β-cell measure was the prespecified primary endpoint: participants on background metformin received tirzepatide titrated to 15 mg, semaglutide 1 mg or placebo for 28 weeks and underwent hyperglycaemic and hyperinsulinaemic-euglycaemic clamps with mixed-meal testing at baseline and week 28 [14]. The total clamp disposition index, which expresses insulin secretion adjusted for prevailing insulin sensitivity and is therefore the most rigorous available index of β-cell competence, increased approximately six-fold from baseline; the trial-reported least-squares mean change was 1.9 (standard error 0.16) with tirzepatide, 1.1 (0.10) with semaglutide and 0.0 (0.03) with placebo. This gain was attributable to concurrent improvement in first-phase, second-phase and total insulin secretion rate and in insulin sensitivity assessed by the M-value, each significantly greater than with semaglutide [14].

**Table 5:** Dynamic and clamp-derived measures of β-cell function and insulin sensitivity AUC: Area under the concentration-time curve; ISR: insulin secretion rate; LS: least squares; SE: standard error. Reports [14] and [18] describe complementary analyses of the same 117 participants.

Study [ref]	Method	Parameter	Tirzepatide result	Comparator	Week
Heise et al. 2022 [14]	Hyperglycaemic and euglycaemic clamp (primary endpoint)	Total clamp disposition index	≈Six-fold rise from baseline; LS mean change 1.9 (SE 0.16)	Semaglutide 1 mg 1.1 (0.10); placebo 0.0 (0.03)	28
Heise et al. 2022 [14]	Hyperglycaemic clamp	Insulin secretion rate (first and second phase)	Significant increase	Greater than semaglutide 1 mg	28
Heise et al. 2022 [14]	Euglycaemic clamp	M-value	Large significant increase vs placebo	Greater than semaglutide 1 mg	28
Mather et al. 2024 [18]	Meal test with model-based analysis	ISR at 7.2 mmol/L glucose	Improved	Exceeded semaglutide 1 mg	28
Mather et al. 2024 [18]	Meal test with model-based analysis	β-cell glucose sensitivity	Improved	Exceeded semaglutide 1 mg	28
Mather et al. 2024 [18]	Meal test	Total and incremental glucose AUC	Total AUC and fasting glucose fell more than with semaglutide (P<0.01); incremental AUC not different	Semaglutide 1 mg	28
Yabe et al. 2023 [15]	Standardised meal test (0-6 h AUC)	Glucose, insulin, C-peptide, glucagon	Significant change vs comparator for all four analytes, all three doses	Dulaglutide 0.75 mg	32
Yamaguchi et al. 2025 [20]	Hyperinsulinaemic-euglycaemic clamp	Glucose infusion rate	1.6-fold rise, 3.21 → 5.16 mg·min^-^¹·kg^-^¹ (P<0.001)	None (single-arm)	12

A subsequent model-based analysis of the meal data from the same trial localised the effect more precisely [18]. Tirzepatide reduced fasting glucose and total meal glucose area under the curve significantly more than semaglutide (P<0.01), whereas the incremental glucose area under the curve did not differ, indicating that the greater total reduction was driven predominantly by superior suppression of fasting glycaemia rather than by attenuation of the postprandial excursion itself. The model-derived parameters, comprising the insulin secretion rate at a fixed glucose concentration of 7.2 mmol/L and β-cell glucose sensitivity, improved with tirzepatide, as did insulin sensitivity and the estimated hepatic insulin-to-glucagon ratio [18].

The pharmacodynamic substudy nested within SURPASS J-mono supplied the only meal-based dynamic data in an East Asian population, with 48 participants allocated to tirzepatide 5, 10 or 15 mg or dulaglutide 0.75 mg [15]. At week 32, the change from baseline in the six-hour area under the concentration-time curve differed significantly from dulaglutide for glucose, insulin, C-peptide, glucagon and triglycerides in all three tirzepatide arms, the sole exception being triglycerides at 10 mg. Reduced postprandial insulin and C-peptide exposure in the presence of markedly lower postprandial glucose again indicates a secretory system operating under substantially reduced demand. In the single-arm study, the clamp demonstrated a 1.6-fold rise in glucose infusion rate from 3.21 to 5.16 mg·min^-^¹·kg^-^¹ after only 12 weeks at a maximum dose of 5 mg (P<0.001), with no significant regression of that change on change in body weight, body composition, HbA1c, lipids or glucagon [20].

α-Cell Function, Insulin Sensitivity, Glycaemia and Body Weight

Ancillary metabolic outcomes are collated in Table 6. Suppression of fasting hyperglucagonaemia was consistent: glucose-adjusted fasting glucagon fell by 37% to 44% against a 4.8% rise on placebo in SURPASS-1 [16], and by 48.7% to 55.3% in SURPASS-2, where the reductions at 10 and 15 mg significantly exceeded the 47.7% achieved with semaglutide [17]; fasting glucagon fell from 28.8 to 20.8 pg/mL in the single-arm study [20], and postprandial glucagon exposure was reduced in both meal-based analyses [15,18]. Insulin sensitivity improved in parallel wherever measured, with HOMA2-IR falling by 9% to 23% against a 14.7% rise on placebo [16] and by 15.5% to 24.0% against a 5.1% fall with semaglutide [17], and HOMA2-%S rising by 21.3% to 49.9% against −8.0% to +8.4% with dulaglutide [19]. Multiple linear regression in the phase 2b analysis indicated that weight loss explained only 13% and 21% of the improvement in HOMA2-IR at the 10 and 15 mg doses (P≤0.028), implying that a substantial component of the insulin-sensitising effect is not attributable to weight reduction alone [13].

**Table 6:** Ancillary outcomes: α-cell function, insulin sensitivity, glycaemia and body weight HbA1c: glycated haemoglobin; HOMA2-IR: homeostatic model assessment index of insulin resistance; HOMA2-%S: homeostatic model assessment index of insulin sensitivity; IGFBP: insulin-like growth factor binding protein. Ancillary outcomes were extracted only from studies that also reported a β-cell function outcome.

Study [ref]	Domain	Tirzepatide	Comparator	Interpretation
Thomas et al. 2021 [13]	Insulin sensitivity, adipokines	HOMA2-IR reduced; adiponectin, IGFBP-1 and IGFBP-2 raised; fasting glucagon reduced	Dulaglutide 1.5 mg and placebo: smaller changes	Weight loss explained only 13% and 21% of the HOMA2-IR gain at 10 and 15 mg (P ≤ .028)
Lee et al. 2023 [16]	α-cell function, insulin sensitivity	Glucose-adjusted glucagon -37% to -44%; HOMA2-IR -9% to -23%; adiponectin +16% to +23%	Placebo: glucagon +4.8%; HOMA2-IR +14.7%; adiponectin -0.2%	Islet and sensitivity gains occur together during monotherapy in early T2DM
Frias et al. 2024 [17]	Glycaemia, weight, α-cell	HbA1c -2.09% to -2.46%; weight -7.8 to -12.4 kg; glucagon -48.7% to -55.3%; HOMA2-IR -15.5% to -24.0%	Semaglutide 1 mg: −1.86%; −6.2 kg; −47.7%; −5.1%	Benefit preserved in every baseline HOMA2-B and HOMA2-IR quartile, no interaction
Mather et al. 2024 [18]	Hepatic and α-cell signalling	Estimated hepatic insulin-to-glucagon ratio improved; greater glucagon suppression	Semaglutide 1 mg: smaller effect	Supports a combined islet and hepatic route to fasting glucose lowering
Hamamoto et al. 2025 [19]	Insulin sensitivity, glycaemia by C-peptide stratum	HOMA2-%S +21.3% to +49.9%; HbA1c -2.31% to -2.89% in both strata	Dulaglutide 0.75 mg: HOMA2-%S -8.0% and +8.4%; HbA1c -1.45% and -1.12%	Glycaemic benefit not conditioned on baseline secretion (interaction P=0.051)
Yamaguchi et al. 2025 [20]	Glycaemia, weight, α-cell	HbA1c 7.95% → 6.14%; weight -4.9 kg (-5.0%); fat mass -9.1%; glucagon 28.8 → 20.8 pg/mL	None (single-arm)	Early effect at 5 mg over 12 weeks, not statistically explained by weight change

These changes were accompanied by the expected glycaemic and anthropometric benefits, and by evidence that benefit does not depend on baseline secretory reserve. In SURPASS-2, HbA1c fell by 2.09% to 2.46% and body weight by 7.8 to 12.4 kg, against 1.86% and 6.2 kg with semaglutide, and the reductions exceeded semaglutide within every baseline HOMA2-B and HOMA2-IR quartile with no quartile-by-treatment interaction [17]. In SURPASS J-mono, HbA1c reductions were significantly greater than with dulaglutide in participants both below and above the median baseline C-peptide of 1.86 µg/L, with a treatment-by-subgroup interaction P value of 0.051 [19]. In the 12-week single-arm study, HbA1c fell from 63.4 to 43.6 mmol/mol (7.95% to 6.14%) and body weight by 4.9 kg, with a 9.1% reduction in fat mass [20].

Risk of Bias

Risk-of-bias judgements are displayed in Figure 3 for the seven reports of randomised trials and in Figure 4 for the single non-randomised study. Four of the seven randomised-trial reports were judged at low risk of bias across all five RoB 2 domains [14,16,18,19], each deriving from a double-blind parent trial with a defined analysis population and objective, centrally assayed outcome measurement. One report raised some concerns overall [15], reflecting concerns in the domains for missing outcome data and for selection of the reported result, consistent with a pharmacodynamic substudy comprising only 48 of 636 participants in unequally sized groups (n=9-19). Two reports were judged at high risk of bias overall [13,17]; in both, the determining domain was selection of the reported result, since the biomarker analyses are described by their authors as post hoc and exploratory, assessed multiple correlated outcomes without adjustment for multiplicity and reported only nominal P values. Both also raised some concerns regarding missing outcome data, and the SURPASS-2 analysis additionally raised concerns regarding deviations from intended interventions because the parent trial was open-label [17], although the objective laboratory nature of the endpoints limits the plausible impact of unblinding. The single-arm interventional study was judged at serious risk of bias overall on ROBINS-I, driven by serious bias due to confounding: without a concurrent control group, the improvement in HOMA2-%β cannot be separated from the concurrent 5.0% weight loss, continued background metformin in some participants or regression to the mean; the remaining domains were judged at low risk apart from moderate concern regarding selection of the reported result [20]. All seven trial-based reports were funded by the manufacturer and most included manufacturer employees among their authors.

**Figure 3:**
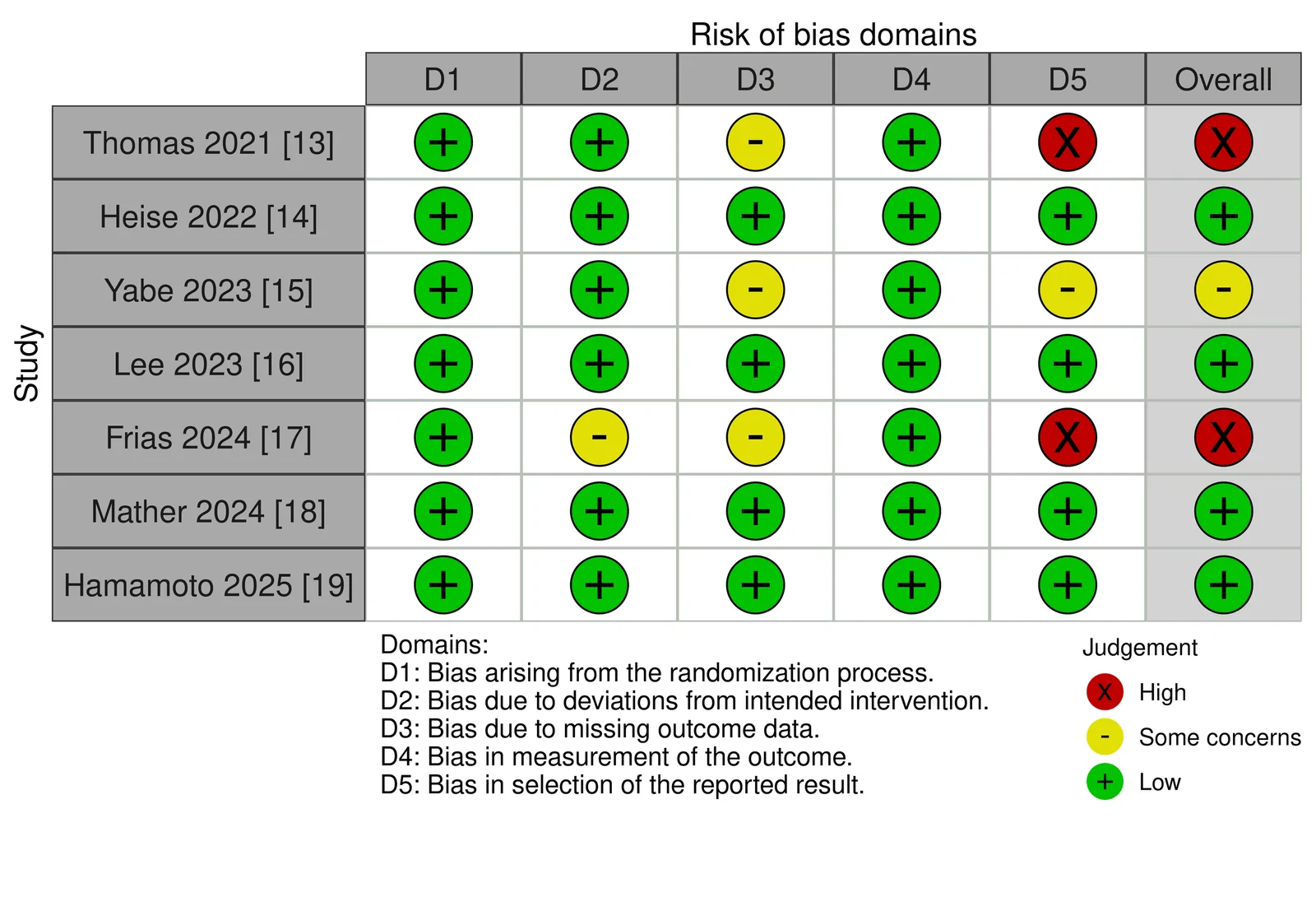
Risk-of-bias summary for the seven reports of randomised controlled trials (RoB 2) Assessment performed with the revised Cochrane risk-of-bias tool for randomised trials (risk of bias 2 (RoB 2)) and visualised with robvis [12]. Judgements are made at the level of the β-cell function outcome rather than the parent trial’s primary endpoint.

**Figure 4:**
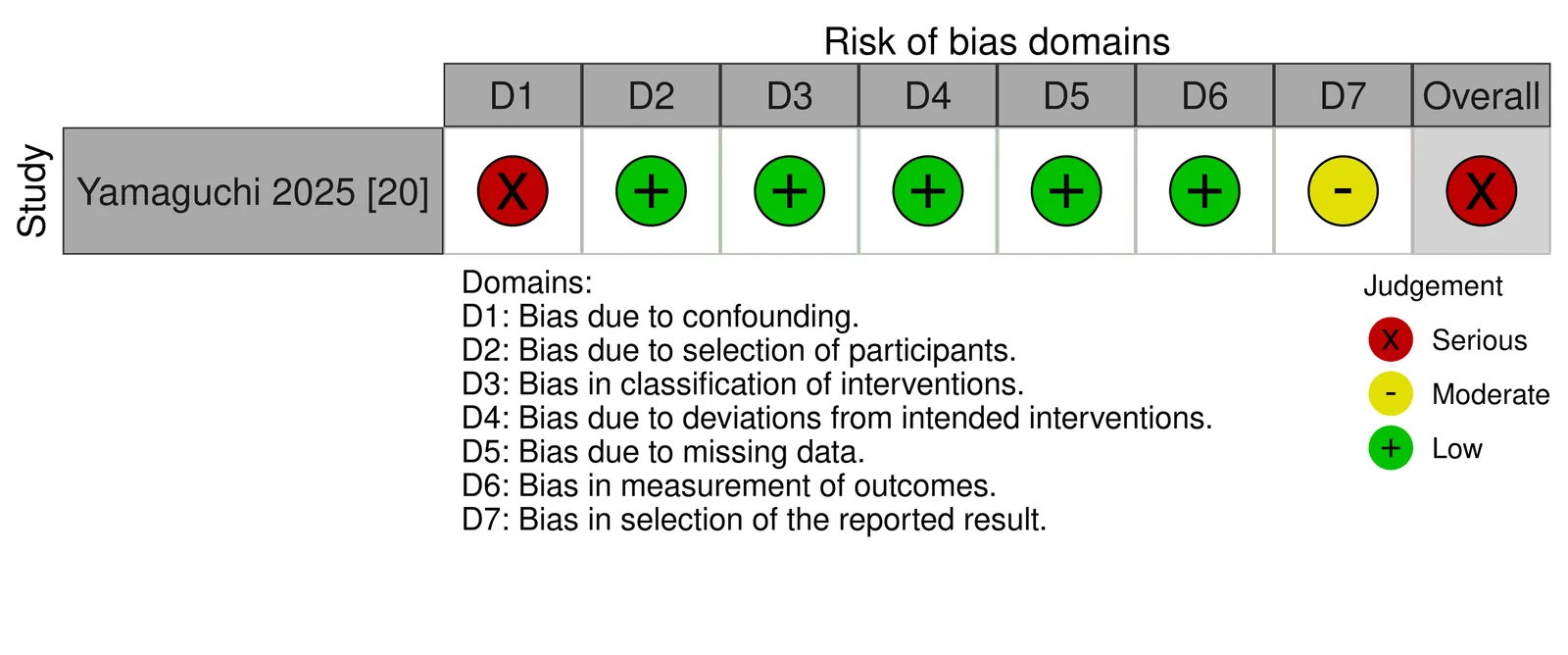
Risk-of-bias summary for the single non-randomised study (ROBINS-I) Assessment performed with the Risk Of Bias In Non-randomised Studies of Interventions (ROBINS-I) tool and visualised with robvis [12]. Judgements are made against the target trial the study emulates, namely a randomised comparison of tirzepatide against no tirzepatide over 12 weeks.

Discussion

Principal Findings

This systematic review assembled eight original peer-reviewed reports, representing six distinct study populations and 3,442 unique participants, that quantified the effect of tirzepatide on pancreatic β-cell function in adults with T2DM. The principal finding is one of striking directional consistency. Across fasting model-derived indices, insulin-processing markers, meal-based model parameters and gold-standard clamp measurements, and across populations that differed substantially in ethnicity, adiposity and background glucose-lowering therapy, tirzepatide improved every index of β-cell function that was measured. Where a comparator was used, the improvement exceeded that achieved with placebo, with dulaglutide at either 0.75 mg or 1.5 mg, and with semaglutide 1 mg, and where multiple maintenance doses were studied, the effect followed a monotonic dose gradient. The consistency of this pattern across methodologically dissimilar approaches is the strongest argument that the observation is real rather than an artefact of any single analytical method.

Interpreting Fasting Indices against Dynamic Measures

The magnitude of the effect on fasting indices deserves emphasis. Increases in HOMA2-B from baseline of 77% to 163% represent an approximate doubling to trebling of the index, and the estimated treatment differences against active comparators, ranging from roughly 7% in the lowest-dose comparison against semaglutide to almost 47% against dulaglutide 0.75 mg, indicate an incremental benefit of dual receptor agonism over selective GLP-1 receptor agonism that is not marginal [17,19]. Yet the interpretation of these numbers requires care, and the second consistent finding of this review supplies the necessary caution. In every study that reported them, fasting insulin and C-peptide concentrations fell rather than rose [16,17,19,20]. HOMA2-B is a glucose-referenced index: it estimates the secretory function required to sustain the observed fasting glucose concentration given the observed insulin or C-peptide concentration. When fasting glucose falls markedly while insulin secretion falls only modestly, the modelled index of β-cell function necessarily rises. What is being demonstrated, therefore, is an improvement in secretory efficiency relative to glycaemic load, and a corresponding reduction in the secretory demand placed upon the β-cell, rather than an expansion of absolute secretory output. This distinction is not a criticism of the evidence but a description of what fasting indices can and cannot establish, and it is precisely why the dynamic and clamp-based reports carry disproportionate evidentiary weight.

Those reports go considerably further. The approximately six-fold increase in the total clamp disposition index observed with tirzepatide 15 mg after 28 weeks, and its clear superiority over semaglutide 1 mg, demonstrates that stimulated insulin secretion adjusted for prevailing insulin sensitivity improved substantially, which cannot be explained by a glucose-referencing artefact [14]. The disposition index is the canonical expression of β-cell competence precisely because it accounts for the hyperbolic relationship between secretion and sensitivity, so that an improvement in sensitivity alone, unaccompanied by any change in secretory capacity, would leave the index unchanged. The subsequent model-based analysis localised the effect further, showing improvement both in the insulin secretion rate at a fixed glucose concentration and in β-cell glucose sensitivity, the parameter describing how briskly the β-cell responds to a rising glucose signal [18]. Taken with reductions in fasting intact proinsulin and the proinsulin/C-peptide ratio of up to 55% and 49%, respectively [13,16], which index the efficiency of proinsulin conversion and are widely interpreted as markers of endoplasmic reticulum stress within the β-cell, the picture that emerges is of a β-cell operating under materially reduced strain and responding more appropriately to glucose.

Mechanistic and Preclinical Evidence

The mechanistic basis of these changes is likely to be composite. Part of the effect is undoubtedly attributable to relief of glucotoxicity and lipotoxicity consequent upon substantial weight loss and improved insulin sensitivity, and the included reports document both. However, the evidence assembled here also indicates that weight loss is an insufficient explanation. Multiple linear regression in the phase 2b analysis attributed only 13% and 21% of the improvement in HOMA2-IR at the 10 mg and 15 mg doses to weight loss [13], and in the 12-week single-arm clamp study the 1.6-fold increase in glucose infusion rate bore no statistically significant relationship to change in body weight, body composition, HbA1c, lipids or glucagon [20]. An exploratory comparison of the phase 1 trial data reported greater improvement in insulin sensitivity per unit of weight lost with tirzepatide than with semaglutide, which is difficult to reconcile with a purely weight-mediated mechanism [21].

Preclinical and in vitro investigations were not eligible for this synthesis because the review question concerned quantified β-cell outcomes in human participants, but that literature is substantial and bears directly on mechanism. Receptor pharmacology studies establish that tirzepatide is an imbalanced dual agonist, engaging the GIP receptor more fully than the GLP-1 receptor and showing biased agonism at the GLP-1 receptor that favours cyclic AMP generation over β-arrestin recruitment, with weaker receptor internalisation than native GLP-1; in primary islets, β-arrestin1 constrained the insulin response to GLP-1 but not to GIP or to tirzepatide, suggesting that this signalling bias itself enhances insulin secretion [22]. In obese mice, GIP receptor agonism mediated insulin sensitisation independently of weight loss, including in animals lacking the GLP-1 receptor [23]. Species differences are material and caution against uncritical extrapolation: in mouse islets, tirzepatide stimulates insulin secretion predominantly through the GLP-1 receptor, owing to reduced potency at the murine GIP receptor, whereas in human islets GIP receptor signalling is required for the hormone-secretory response to the drug [24]. Beyond the islet, long-acting GIP receptor activation remodels adipocyte nutrient handling, sustaining lipid storage capacity and fatty acid oxidation in a manner expected to reduce ectopic lipid delivery to the islet [25]. Taken together, this work supplies a coherent biological substrate for the clinical observations synthesised here, and the human-islet data in particular support the view that the GIP receptor component contributes materially to the secretory improvement. A β-cell exposed to lower ambient glucose, lower non-esterified fatty acid flux and lower insulin demand has both less stimulus for maximal secretion and less exposure to the stressors that drive dedifferentiation. These mechanistic data cannot, however, substitute for the human withdrawal studies identified below as the critical evidence gap.

Comparison with Previous Evidence

These findings sit coherently within the wider literature on incretin therapy and β-cell biology. The parent SURPASS trials established the clinical phenotype that this review seeks to explain, with tirzepatide producing HbA1c reductions substantially greater than insulin glargine in a high-cardiovascular-risk population over two years [26] and greater than dulaglutide 0.75 mg over 52 weeks in Japanese participants [27]. The present synthesis suggests that a meaningful part of that clinical superiority is mediated through islet-level effects rather than through weight loss alone. A complementary line of evidence comes from populations without diabetes: a post hoc analysis of the SURMOUNT-1 trial in 2,539 adults with obesity or overweight and either prediabetes or normoglycaemia found that 72 weeks of tirzepatide improved oral glucose tolerance test-derived measures of both β-cell function and insulin sensitivity across all doses and in both glycaemic strata, with improvements only partly explained by weight change [28]. That study was excluded from the present review because its population did not have T2DM, but its findings are directly relevant, indicating that the islet effects of tirzepatide are not contingent on established diabetes and raising the possibility that the drug acts to prevent the deterioration of β-cell function as well as to improve it once impaired.

Clinical Implications

Two clinically actionable observations emerge from the included evidence. The first concerns patient selection. Both the SURPASS-2 quartile analysis and the SURPASS J-mono subgroup analysis addressed whether baseline β-cell reserve predicts response, and both concluded that it does not in any clinically limiting way. Glycaemic and weight benefits exceeded those of the active comparator within every baseline HOMA2-B and HOMA2-IR quartile, with no significant quartile-by-treatment interaction [17], and HbA1c reductions were significantly greater than with dulaglutide in participants both below and above the median baseline C-peptide, with an interaction P value of 0.051 [19]. The largest absolute HbA1c reductions were in fact observed in the lowest HOMA2-B quartile, although this is at least partly explained by the substantially higher baseline HbA1c in that group [17]. Clinicians should therefore not withhold tirzepatide on the presumption that diminished endogenous secretory reserve will blunt the response. The second observation concerns timing. The largest proportional improvements in fasting β-cell indices were seen in the monotherapy settings enrolling participants with shorter diabetes duration and no background pharmacotherapy [16,19], consistent with the principle that the β-cell is more amenable to functional rescue earlier in the disease course, before secretory capacity has been irrecoverably lost.

Knowledge Gaps and Future Research

Several knowledge gaps define the agenda for future work. The most consequential is that no included study assessed β-cell function after the withdrawal of tirzepatide. Every reported improvement is an on-treatment observation, and on-treatment improvement in a glucose-referenced or demand-referenced index is fully compatible with a purely pharmacological effect that reverses on discontinuation. Distinguishing genuine disease modification, meaning a durable alteration in the trajectory of β-cell decline, from sustained pharmacological compensation requires studies incorporating a washout period of sufficient duration to eliminate drug effect, followed by repeat clamp or meal-based assessment; until such a study is performed, claims that tirzepatide preserves or restores β-cell function should be framed carefully. Second, no human study has measured β-cell mass or examined β-cell identity directly, so it remains unknown whether the functional improvements reflect recovery of dedifferentiated cells, improved function of existing cells, or both. Third, the longest follow-up for a β-cell outcome was 52 weeks, which is short relative to the multi-decade natural history of the secretory defect. Fourth, the evidence base is geographically constrained: three of the six study populations were exclusively Japanese and the remainder predominantly of European or North American origin, with no dedicated analyses in populations of African, South Asian or Latin American ancestry, in whom the relative contributions of secretory failure and insulin resistance differ. Fifth, no included study examined β-cell outcomes in participants with long-standing insulin-treated diabetes, in whom the therapeutic question is arguably most pressing. Finally, every included report was funded by the manufacturer and most listed manufacturer employees as authors; independent replication of the clamp-based findings, which currently rest on a single trial of 117 participants reported in two complementary papers [14,18], would materially strengthen the evidence.

Methodological Considerations

The methodological character of this evidence base also warrants comment. Only one of the eight included reports had a β-cell function measure as a prespecified primary endpoint [14]. The remainder were prespecified secondary analyses, post hoc exploratory biomarker analyses or subgroup analyses, and three were judged at high or serious risk of bias, in each case driven by the domain concerning selection of the reported result or, for the non-randomised study, by confounding. Post hoc analyses conducted without adjustment for multiplicity, reporting nominal P values across many correlated biomarkers, are vulnerable to selective emphasis even when the underlying trial was rigorously conducted. This does not invalidate the findings, particularly given their consistency and the biological coherence of the pattern, but it does mean that the confidence attaching to the precise magnitude of individual estimates should be lower than the confidence attaching to the direction and general size of the effect. The decision not to pool these data quantitatively was made deliberately for the same reason: combining a clamp-derived disposition index, a C-peptide-based homeostatic model index and a meal-derived secretion rate into a single standardised mean difference would confer a spurious appearance of precision on measurements that answer different physiological questions, and the overlapping participant populations across four of the eight reports would violate the independence assumption on which pooling rests.

Limitations

This review has several limitations that qualify its conclusions. Screening, data extraction and risk-of-bias appraisal were not performed in duplicate independently by two or more assessors, which is the standard safeguard against selection and extraction error in systematic reviews; the duplicate-pass procedure, data verification, re-run search and citation tracking described in the Methods mitigate but do not eliminate that risk, and readers should weigh the findings accordingly. The protocol was not prospectively registered. Eligibility was restricted to English-language full texts; although no record was in fact excluded on language grounds in this review, the restriction could matter for future updates given the concentration of relevant research in Japan. Quantitative synthesis was not possible, so no pooled effect estimate, no formal assessment of statistical heterogeneity and no evaluation of small-study effects could be undertaken, and the certainty of the body of evidence was not graded using GRADE. Preclinical and in vitro studies were excluded by design, so the mechanistic literature discussed above was not systematically searched or appraised and is cited illustratively rather than exhaustively. The evidence base itself imposes further constraints: it is small, uniformly industry-funded, concentrated in the SURPASS clinical development programme, and dominated by post hoc analyses in which β-cell function was an exploratory rather than a primary endpoint. Four of the eight reports describe participants drawn from only two parent trials, so the apparent breadth of the evidence exceeds its true independence. The longest follow-up was 52 weeks, and no study examined β-cell outcomes after treatment cessation, in insulin-treated populations, or in populations of African, South Asian or Latin American ancestry.

## Conclusions

Across eight original studies encompassing six distinct populations and 3,442 adults with type 2 diabetes mellitus, once-weekly subcutaneous tirzepatide consistently improved indices of pancreatic β-cell function. Fasting model-derived indices approximately doubled to tripled, markers of β-cell secretory stress fell by roughly half, and the clamp disposition index rose approximately six-fold, in each case exceeding the improvement achieved with placebo or with selective GLP-1 receptor agonism, and in a dose-dependent manner. These gains were accompanied by suppression of fasting and postprandial hyperglucagonaemia and by improved insulin sensitivity that was only partly attributable to weight loss, and they were evident irrespective of baseline β-cell reserve. Tirzepatide can therefore be regarded as improving the physiological function of the pancreatic islet in type 2 diabetes, and not merely as lowering glucose. Whether this on-treatment improvement translates into durable modification of the natural history of β-cell failure remains unresolved, because no study has assessed β-cell function after drug withdrawal; adequately powered trials incorporating a washout period and dynamic secretory testing, conducted in more diverse populations and independently of the manufacturer, are the necessary next step.
